# Analytical Solutions for Multi-Time Scale Fractional Stochastic Differential Equations Driven by Fractional Brownian Motion and Their Applications

**DOI:** 10.3390/e20010063

**Published:** 2018-01-16

**Authors:** Xiao-Li Ding, Juan J. Nieto

**Affiliations:** 1 Department of Mathematics, Xi’an Polytechnic University, Xi’an 710048, China; 2 Departamento de Estatística, Análisis Matemático y Optimización, Universidad de Santiago de Compostela, 15782 Santiago de Compostela, Spain

**Keywords:** multi-time scale fractional stochastic differential equations, fractional Brownian motion, fractional stochastic partial differential equation, analytical solution

## Abstract

In this paper, we investigate analytical solutions of multi-time scale fractional stochastic differential equations driven by fractional Brownian motions. We firstly decompose homogeneous multi-time scale fractional stochastic differential equations driven by fractional Brownian motions into independent differential subequations, and give their analytical solutions. Then, we use the variation of constant parameters to obtain the solutions of nonhomogeneous multi-time scale fractional stochastic differential equations driven by fractional Brownian motions. Finally, we give three examples to demonstrate the applicability of our obtained results.

## 1. Introduction

In the last few years, the interest of the scientific community towards fractional calculus has experienced an exceptional boost, and so its applications can now be found in a great variety of scientific fields—for example, anomalous diffusion [1,2,3], medicine [4], solute transport [5], random and disordered media [6,7,8], information theory [9], electrical circuits [10], and so on. The reason for the success of fractional calculus in modeling natural phenomena is that the operators are nonlocal, which makes them suitable to describe the long memory or nonlocal effects characterizing most physical phenomena.

Fractional stochastic differential equations (FSDEs) are an important class of differential equations. They can model the dynamics of complex systems in finance [8,11,12,13], and in physical problems [14,15]. For example, in [8], the authors combined stochastic contact process and compound Poisson process to construct a novel microscope complex price dynamics, in an attempt to reproduce and characterize the complex dynamics of financial markets. In finance, fractional permutation entropy, sample entropy, and fractional sample entropy play important roles. It is well-known that entropy is used to quantify the complexity and uncertainty in financial time series and others. At the same time, the necessity of a powerful technique for solving these new types of equations arose, becoming one of the main research objects in the fields of theoretical and applied sciences. In the available literature, there exist various methods for solving fractional stochastic differential equations, such as analytical methods and numerical algorithms [16,17,18,19,20,21,22,23,24,25,26,27,28,29,30,31].

Analytical solutions of fractional partial equations are of fundamental importance in describing and understanding physical phenomena, since all the parameters are expressed in the form of infinite series, and therefore the influence of individual parameters on natural phenomena can be easily examined. Additionally, the analytical solutions make it easy to study asymptotic behaviors of the solutions, which are usually difficult to obtain through numerical calculations. Besides, the analytical solutions may serve as tools in assessing the computational performance and accuracy of numerical solutions. Especially, for stochastic differential systems, analytical solutions may provide a useful tool for assessing the influence of some parameters on statistical properties, permutation entropy, fractional permutation entropy, sample entropy, and fractional sample entropy. It is well-known that entropy theory is an important issue because it enables hydraulic and control engineers to quantify uncertainties, determine risk and reliability, estimate parameters, model processes, and design more robust and reliable hydraulic canals control systems.

To the authors’ knowledge, the analytical solutions of the FSDEs driven by fractional Brownian motions (fBms) have not yet been reported in the literature. X.J. Wang et al. [32] considered the following semilinear parabolic SPDEs in V, driven by an infinite dimensional fractional Brownian motion,

(1) $$\begin{array}{c} dX\left(t\right)+AX\left(t\right)dt=F\left(X\left(t\right)\right)dt+\Phi dB_{H}\left(t\right),X\left(0\right)=x_{0},t\in \left[0,T\right], \end{array}$$

where F:V→V and Φ:V→V are deterministic mappings. Motivated by their work, we investigate the analytical solution of the following multi-time scale fractional stochastic differential equation:

(2) $$\begin{array}{c} \frac{dY(t)}{dt}+D_{t}^{\alpha }\left(a(t)Y(t)+p(t)\right)=\left(b(t)Y(t)+q(t)\right)+\left(\sigma (t)Y(t)+v(t)\right)\frac{dB_{H}\left(t\right)}{dt},Y\left(0\right)=y_{0}, \end{array}$$

where b,p,σ,q,a,v∈C([0,T]), 0<α≤1, $B_{H}$ is a fractional Brownian motion defined on [0,T], and $y_{0}$ is a real-valued random variable on a complete probability space (Ω,F,P), and it is independent of $B_{H}\left(t\right)$ for all t∈[0,T]. The detailed definitions of the Riemann–Liouville fractional derivative and the fractional Laplacian operator and fBm are given in the next section (or see [33,34,35,36,37]).

The rest of this paper is organized as follows. In Section 2, we give some basic definitions of fBm and fractional calculus, which will be used in the paper. In Section 3, we give the solution of multi-time scale FSDEs driven by fBms. In Section 4, we give three examples to demonstrate the applicability of the obtained results. In Section 5, we give conclusions.

## 2. Preliminaries

In this section, we give some basic definitions of fractional Brownian motion and fractional calculus, which will be used throughout this paper. For details, one can refer to [37,38,39,40].

Let (Ω,F,P) be a complete probability space, and [0,T] be a finite time interval.

**Definition** **1.** *A one-dimensional fractional Brownian motion $B_{H}=\left\{B_{H}\left(t\right),t\in \left[0,T\right]\right\}$ of Hurst index H∈(0,1) on [0,T] is a continuous and centered Gaussian process on some probability space (Ω,F,P) with covariance function*

$$E\left[B_{H}\left(t\right)B_{H}\left(s\right)\right]=\frac{1}{2}(t^{2H}+s^{2H}{-|t-s|}^{2H}),\;t,s\in \left[0,T\right].$$

If $H=\frac{1}{2}$, then the corresponding fBm is the usual standard Brownian motion. If $H>\frac{1}{2}$, then the process fBm exhibits a long-range dependence. In this paper, we always assume $H\in (\frac{1}{2},1)$.

**Lemma** **1.** *(Fractional Itô formula) [39] If X(t) satisfies that*

(3) $$\begin{array}{c} dX\left(t\right)=u\left(t\right)dt+v\left(t\right)dB_{H}\left(t\right), \end{array}$$

*where u,v are given functions. Furthermore, let $f\in C^{2}\left(R\right)$, and assume that $f^{\prime }\left(X\right)$ and $f^{\prime \prime }\left(X\right)$ exist and are continuous for X∈R. Then, it has*

(4) $$\begin{array}{c} df\left(X\left(t\right)\right)=\left(f^{\prime }\left(X\left(t\right)\right)u\left(t\right)+Hf^{\prime \prime }\left(X\left(t\right)\right)t^{2H-1}v^{2}\left(t\right)\right)dt+f^{\prime }\left(X\left(t\right)\right)v\left(t\right)dB_{H}\left(t\right). \end{array}$$

It is interesting to note that if $H=\frac{1}{2}$ is formally substituted in Equation (4), then the well-known Itô formula for classical Brownian motion is obtained.

In the following, we recall some definitions about fractional calculus and some special functions.

**Definition** **2.** *Let α>0. Then, the Riemann–Liouville fractional integral of order α with respect to t is defined as*

(5) $$\begin{array}{c} I_{t}^{\alpha }f\left(t\right)=\frac{1}{\Gamma (\alpha )}\int_{0}^{t}{\left(t-\tau \right)}^{\alpha -1}f\left(\tau \right)d\tau ,\;t>0, \end{array}$$

*where Γ(·) is the Gamma function.*

**Definition** **3.** *Let f∈C([0,T]) and m−1<α≤m, where $m\in N^{+}$. The Riemann–Liouville fractional derivative of order α with respect to t is defined as*

(6) $$\begin{array}{c} D_{t}^{\alpha }f\left(t\right)=\frac{1}{\Gamma (m-\alpha )}\frac{d^{m}}{dt^{m}}\int_{0}^{t}{\left(t-\tau \right)}^{m-\alpha -1}f\left(\tau \right)d\tau ,\;t>0. \end{array}$$

There exists the following relationship between the Riemann–Liouville fractional integral and the Riemann–Liouville fractional derivative.

**Property** **1.** *Let m−1<α≤m, where $m\in N^{+}$ [37]. Then the statements are true:*

(7) $$\begin{array}{c} \left(D_{t}^{\alpha }I_{t}^{\alpha }f\right)\left(t\right)=f\left(t\right),\;\left(I_{t}^{\alpha }D_{t}^{\alpha }f\right)\left(t\right)=f\left(t\right)-\sum_{k=1}^{m}\frac{{\left(I_{t}^{m-\alpha }f\right)}^{\left(m-k\right)}\left(0^{+}\right)}{\Gamma (\alpha -k+1)}t^{\alpha -k},\;t>0. \end{array}$$

**Definition** **4.** *Suppose that the Laplacian (−▵) has a complete set of orthonormal eigenfunctions $\varphi_{n}$ corresponding to eigenvalues $\lambda_{n}^{2}$ on a bounded region D; i.e., $\left(-▵\right)\varphi_{n}=\lambda_{n}^{2}\varphi_{n}$ on D; $B(\varphi_{n})=0$ on ∂D, where $B(\varphi_{n})$ is one of the standard three homogeneous boundary conditions [33]. Let*

(8) $$G=\{g=\sum_{n=1}^{\infty }c_{n}\varphi_{n},\;c_{n}=\langle g,\varphi_{n}\rangle ,\;\sum_{n=1}^{\infty }|c_{n}{|}^{2}{\left|\lambda_{n}\right|}^{\alpha }<\infty \},$$

*then for any g∈G, ${\left(-▵\right)}^{\frac{\alpha }{2}}$ is defined by*

(9) $$\begin{array}{c} {\left(-▵\right)}^{\frac{\alpha }{2}}g=\sum_{n=1}^{\infty }c_{n}\lambda_{n}^{\alpha }\varphi_{n}. \end{array}$$

**Lemma** **2.** Suppose that the one-dimensional Laplacian (−▵) defined with Dirichlet boundary conditions at x=0 and x=L has a complete set of orthonormal eigenfunctions $\varphi_{n}$ corresponding to eigenvalues $\lambda_{n}^{2}$ on a bounded region [0,L] [33]. If $\left(-▵\right)\varphi_{n}=\lambda_{n}^{2}\varphi_{n}$ on [0,L], and $\varphi_{n}\left(0\right)=\varphi_{n}\left(L\right)=0$, then, the eigenvalues are given by $\lambda_{n}^{2}=\frac{n^{2}\pi^{2}}{L^{2}}$, and the corresponding eigenfunctions are $\varphi_{n}\left(x\right)=\sin\left(n\pi x/L\right)$, n=1,2,….

**Definition** **5.** *The two-parameter Mittag–Leffler function is defined by [37]*

(10) $$\begin{array}{c} E_{\alpha ,\beta }\left(z\right)=\sum_{k=0}^{\infty }\frac{z^{k}}{\Gamma (\alpha k+\beta )},\;\alpha ,\beta >0. \end{array}$$

*The one-parameter Mittag–Leffler function is defined by*

(11) $$\begin{array}{c} E_{\alpha }\left(z\right)=\sum_{k=0}^{\infty }\frac{z^{k}}{\Gamma (\alpha k+1)},\;\alpha >0. \end{array}$$

In particular, when β=1, the two-parameter Mittag–Leffler function coincides with the one-parameter Mittag–Leffler function; i.e., $E_{\alpha ,1}\left(z\right)=E_{\alpha }\left(z\right)$.

**Definition** **6.** *A generalized Mittag–Leffler function is defined by [37]*

(12) $$\begin{array}{c} E_{\alpha ,m,l}\left(z\right)=\sum_{k=0}^{\infty }c_{k}z^{k}, \end{array}$$

*with*

(13) $$\begin{array}{c} c_{0}=1,\;c_{k}=\prod_{j=0}^{k-1}\frac{\Gamma (\alpha (jm+1))}{\Gamma (\alpha (jm+l+1)+1)}, \end{array}$$

*where α>0, m>0, and α(jm+l)>0.*

In particular, when m=1, there exists the following relationship between the generalized Mittag–Leffler function and the two-parameter Mittag–Leffler function:

(14) $$\begin{array}{c} E_{\alpha ,1,l}\left(z\right)=\Gamma \left(\alpha l+1\right)E_{\alpha ,\alpha l+1}\left(z\right). \end{array}$$

## 3. Solution Representation for FSDEs Driven by fBms

In this section, we first give an equivalent form of Equation (2) and then investigate its analytical solution. Before giving its equivalent form, we provide some explanations about the Riemann–Liouville fractional integral. In [29] (Definition 3.2 and Example 3.1), the authors gave that the integral with respect to ${\left(dt\right)}^{\alpha }$ defined as

(15) $$\begin{array}{c} \int_{0}^{t}f\left(\tau \right){\left(d\tau \right)}^{\alpha }=\alpha \int_{0}^{t}{\left(t-\tau \right)}^{\alpha -1}f\left(\tau \right)d\tau ,\;t>0, \end{array}$$

where f∈C([0,T]) and 0<α≤1. Based on this definition, we can obtain the following relationship between the Riemann–Liouville fractional integral and the integral with respect to ${\left(dt\right)}^{\alpha }$:

(16) $$\begin{array}{c} \int_{0}^{t}f\left(\tau \right){\left(d\tau \right)}^{\alpha }=\alpha \Gamma \left(\alpha \right)\left(I_{t}^{\alpha }f\right)\left(t\right).\;t>0, \end{array}$$

where f∈C([0,T]) and 0<α≤1.

One sees that Equation (2) is equivalent to the following integral equation:

(17) $$\begin{array}{ccc} Y(t) & = & y_{0}-\frac{1}{\Gamma (1-\alpha )}\int_{0}^{t}{\left(t-\tau \right)}^{-\alpha }\left(a(\tau )Y(\tau )+p(\tau )\right)d\tau +\int_{0}^{t}\left(b(\tau )Y(\tau )+q(\tau )\right)d\tau \\ & + & \int_{0}^{t}\left(\sigma (\tau )Y(\tau )+v(\tau )\right)dB_{H}\left(\tau \right). \end{array}$$

By (15), the above equation can be rewritten as:

(18) $$\begin{array}{ccc} Y(t) & = & y_{0}-\frac{1}{\Gamma (2-\alpha )}\int_{0}^{t}\left(a(\tau )Y(\tau )+p(\tau )\right){\left(d\tau \right)}^{1-\alpha }+\int_{0}^{t}\left(b(\tau )Y(\tau )+q(\tau )\right)d\tau \\ & + & \int_{0}^{t}\left(\sigma (\tau )Y(\tau )+v(\tau )\right)dB_{H}\left(\tau \right). \end{array}$$

That is to say, Equation (2) is equivalent to the following equation:

(19) $$\left\{\begin{array}{c} dY\left(t\right)=\frac{1}{\Gamma (2-\alpha )}\left(a(t)Y(t)+p(t)\right){\left(dt\right)}^{1-\alpha }+\left(b(t)Y(t)+q(t)\right)dt+\left(\sigma (t)Y(t)+v(t)\right)dB_{H}\left(t\right),\\ Y\left(0\right)=y_{0}. \end{array}\right.$$

Therefore, we only need to solve Equation (19). For obtaining the solution of Equation (19), we first discuss the solution of the correspondent homogeneous case in the next subsection.

### 3.1. Solution Representation for Linear Homogeneous Case

The corresponding homogeneous differential equation can be written as:

(20) $$\left\{\begin{array}{c} dY\left(t\right)=\frac{1}{\Gamma (2-\alpha )}a\left(t\right)Y\left(t\right){\left(dt\right)}^{1-\alpha }+b\left(t\right)Y\left(t\right)dt+\sigma \left(t\right)Y\left(t\right)dB_{H}\left(t\right),\\ Y\left(0\right)=y_{0}. \end{array}\right.$$

To obtain the solution of Equation (20), we decompose Equation (20) into three subequations:

(21) $$\begin{array}{c} dY^{f}\left(t\right)=\frac{1}{\Gamma (2-\alpha )}a\left(t\right)Y^{f}\left(t\right){\left(dt\right)}^{1-\alpha },\;Y^{f}\left(0\right)=y_{0}^{f}, \end{array}$$

(22) $$\begin{array}{c} dY^{d}\left(t\right)=b\left(t\right)Y^{d}\left(t\right)dt,\;Y^{d}\left(0\right)=y_{0}^{d}, \end{array}$$

(23) $$\begin{array}{c} dY^{s}\left(t\right)=\sigma \left(t\right)Y^{s}\left(t\right)dB_{H}\left(t\right),\;Y^{s}\left(0\right)=y_{0}^{s}, \end{array}$$

where $y_{0}^{f},y_{0}^{d},y_{0}^{s}$ are constants which satisfy $y_{0}^{f}y_{0}^{d}y_{0}^{s}=y_{0}$. Obviously, we have

(24) $$\begin{array}{ccc} d(Y^{f}Y^{d}Y^{s}) & = & Y^{d}Y^{s}\left(dY^{f}\right)+Y^{f}Y^{s}\left(dY^{d}\right)+Y^{f}Y^{d}\left(dY^{s}\right) \end{array}$$

(25) $$\begin{array}{cc} = & Y^{d}Y^{s}\frac{1}{\Gamma (2-\alpha )}a\left(t\right)Y^{f}\left(t\right){\left(dt\right)}^{1-\alpha }+Y^{f}Y^{s}b\left(t\right)Y^{d}\left(t\right)dt+Y^{f}Y^{d}\sigma \left(t\right)Y^{s}\left(t\right)dB_{H}\left(t\right) \end{array}$$

(26) $$\begin{array}{cc} = & \frac{1}{\Gamma (2-\alpha )}a\left(t\right)Y\left(t\right){\left(dt\right)}^{1-\alpha }+b\left(t\right)Y\left(t\right)dt+\sigma \left(t\right)Y\left(t\right)dB_{H}\left(t\right). \end{array}$$

This implies that $Y=Y^{f}Y^{d}Y^{s}$ is the solution of Equation (20).

In the following, our aim is to solve Equations (21) and (23), because the solution of Equation (22) is well-known. Firstly, we consider the solution of Equation (21).

**Lemma** **3.** *Let 0<α<1 and a∈C([0,T]). Then, the solution of Equation* (21) *is given by*

(27) $$\begin{array}{c} Y^{f}\left(t\right)=\sum_{i=0}^{\infty }R_{a}^{i}y_{0}^{f}, \end{array}$$

*where $R_{a}$ is an operator defined on C([0,T]):*

(28) $$\begin{array}{c} \left(R_{a}\varphi \right)\left(t\right)=\frac{1}{\Gamma (1-\alpha )}\int_{0}^{t}{\left(t-\tau \right)}^{-\alpha }a\left(\tau \right)\varphi \left(\tau \right)d\tau , \end{array}$$

*and $R_{a}^{0}$ is an identity operator, and $R_{a}^{i}$ denotes the i-times composition operator of $R_{a}$, i=1,2,….*

**Proof.**  Note that Equation (21) is equivalent to the following integral equation:

(29) $$\begin{array}{c} Y\left(t\right)=y_{0}+\frac{1}{\Gamma (1-\alpha )}\int_{0}^{t}{\left(t-\tau \right)}^{-\alpha }a\left(\tau \right)Y\left(\tau \right)d\tau . \end{array}$$

Construct a successive approximate sequence $\left\{Y_{\left(k\right)}^{f}\right\}$ defined as:

(30) $$\begin{array}{c} Y_{\left(k+1\right)}^{f}\left(t\right)=y_{0}+\frac{1}{\Gamma (1-\alpha )}\int_{0}^{t}{\left(t-\tau \right)}^{-\alpha }a\left(\tau \right)Y_{\left(k\right)}^{f}\left(\tau \right)d\tau ,\;k=0,1,2,\ldots , \end{array}$$

where we choose $Y_{\left(0\right)}^{f}\left(t\right)\equiv y_{0}^{f}$. Then, by induction on *k*, we can obtain

(31) $$\begin{array}{c} Y_{\left(k\right)}^{f}\left(t\right)=\sum_{i=0}^{k}R_{a}^{i}y_{0}^{f},\;k=0,1,2,\ldots , \end{array}$$

where the operator $R_{a}$ is defined in (28).

Next, we will show that the series $\sum_{i=0}^{\infty }\left(R_{a}^{i}y_{0}^{f}\right)\left(t\right)$ is uniformly convergent with respect to t∈[0,T]. Because a(t)∈C([0,T]), there exists M>0 such that ∥a∥≤M for any t∈[0,T]. Based on this consideration, we have

(32) $$\begin{array}{c} ∥\left(R_{a}y_{0}^{f}\right)\left(t\right)∥=∥\frac{y_{0}^{f}}{\Gamma (1-\alpha )}\int_{0}^{t}{\left(t-\tau \right)}^{-\alpha }a\left(\tau \right)d\tau ∥\leq \frac{y_{0}^{f}Mt^{1-\alpha }}{\Gamma (2-\alpha )}. \end{array}$$

Furthermore, suppose that the following relationship

(33) $$\begin{array}{c} ∥\left(R_{a}^{i}y_{0}^{f}\right)\left(t\right)∥\leq \frac{y_{0}^{f}M^{i}t^{i(1-\alpha )}}{\Gamma (i(1-\alpha )+1)} \end{array}$$

holds for any fixed i∈N. Let us prove that the relationship (33) is also valid for i+1. According to the induction hypothesis, we get

(34) $$\begin{array}{ccc} ∥\left(R_{a}^{i+1}y_{0}^{f}\right)\left(t\right)∥ & = & \frac{1}{\Gamma (1-\alpha )}∥\int_{0}^{t}{\left(t-\tau \right)}^{-\alpha }a\left(\tau \right)\left(R_{a}^{i}y_{0}^{f}\right)\left(\tau \right)d\tau ∥ \end{array}$$

(35) $$\begin{array}{cc} \leq & \frac{y_{0}^{f}M^{i+1}}{\Gamma (1-\alpha )\Gamma (i(1-\alpha )+1)}\int_{0}^{t}{\left(t-\tau \right)}^{-\alpha }\tau^{i(1-\alpha )}d\tau . \end{array}$$

Making use of a variable substitution τ=ωt, we have

(36) $$\begin{array}{c} \int_{0}^{t}{\left(t-\tau \right)}^{-\alpha }\tau^{i(1-\alpha )}d\tau =t^{\left(i+1)(1-\alpha \right)}\int_{0}^{1}{\left(1-\omega \right)}^{-\alpha }\omega^{i(1-\alpha )}d\omega =t^{\left(i+1)(1-\alpha \right)}\frac{\Gamma (1-\alpha )\Gamma (i(1-\alpha )+1)}{\Gamma ((i+1)(1-\alpha )+1)}, \end{array}$$

where B(·,·) is the Beta function defined as

(37) $$\begin{array}{c} B\left(z,w\right)=\int_{0}^{1}{\left(1-\tau \right)}^{z-1}\tau^{w-1}d\tau ,z,w>0. \end{array}$$

Here we used the relationship between the Beta function and the Gamma function:

(38) $$\begin{array}{c} B\left(z,w\right)=\frac{\Gamma (z)\Gamma (w)}{\Gamma (z+w)}. \end{array}$$

So it has $∥\left(R_{a}^{i+1}y_{0}^{f}\right)\left(t\right)∥\leq \frac{y_{0}^{f}M^{i+1}t^{\left(i+1)(1-\alpha \right)}}{\Gamma ((i+1)(1-\alpha )+1)}$. Hence, for any i∈N, we have

(39) $$\begin{array}{c} ∥\left(R_{a}^{i}y_{0}^{f}\right)\left(t\right)∥\leq \frac{y_{0}^{f}M^{i}t^{i(1-\alpha )}}{\Gamma (i(1-\alpha )+1)}. \end{array}$$

That is to say, the series $\sum_{i=0}^{\infty }\left(R_{a}^{i}y_{0}^{f}\right)\left(t\right)$ is uniformly convergent with respect to t∈[0,T], and the sum function is the unique solution of Equation (21). This completes the proof of this lemma. ☐

With respect to this lemma, we have the following remarks.

**Remark** **1.** *In particular, if α=0, then we have*

(40) $$\begin{array}{c} \left(R_{a}^{i}y_{0}^{f}\right)\left(t\right)=\frac{y_{0}^{f}{\left(\int_{0}^{t}a\left(\tau \right)d\tau \right)}^{i}}{i!},\;i=1,2,\ldots . \end{array}$$

*Obviously*, (40) *is valid for i=1. Suppose that* (40) *holds for any fixed i. Let us verify that* (40) *also holds for i+1. According to the induction hypothesis, we have*

(41) $$\begin{array}{c} \left(R_{a}^{i+1}y_{0}^{f}\right)\left(t\right)=y_{0}^{f}\int_{0}^{t}a\left(\tau \right)\frac{{\left(\int_{0}^{\tau }a\left(s\right)ds\right)}^{i}}{i!}d\tau =y_{0}^{f}\int_{0}^{t}\frac{{\left(\int_{0}^{\tau }a\left(s\right)ds\right)}^{i}}{i!}d\left(\int_{0}^{\tau }a\left(s\right)ds\right)=\frac{y_{0}^{f}{\left(\int_{0}^{t}a\left(\tau \right)d\tau \right)}^{i+1}}{(i+1)!}. \end{array}$$

*So*, (40) *holds for any positive integer. Therefore, the solution of the following initial value problem*

(42) $$\begin{array}{c} \frac{dY^{f}\left(t\right)}{dt}=a\left(t\right)Y^{f}\left(t\right),\;Y^{f}\left(0\right)=y_{0}^{f} \end{array}$$

*is given by*

(43) $$\begin{array}{c} Y^{f}\left(t\right)=y_{0}^{f}\exp\left(\int_{0}^{t}a\left(\tau \right)d\tau \right). \end{array}$$

This coincides with the classical result.

**Remark** **2.** *In [29], the author gave the solution of Equation* (21) *as*

(44) $$\begin{array}{c} Y^{f}\left(t\right)=y_{0}^{f}E_{1-\alpha }\left(\left(1-\alpha \right)\int_{0}^{t}{\left(t-\tau \right)}^{-\alpha }a\left(\tau \right)d\tau \right). \end{array}$$

*We think the representation of the solution of Equation* (21) *is wrong. For example, $\alpha =\frac{1}{2}$, and $a\left(t\right)=t^{\beta }$, it has*

(45) $$\begin{array}{c} \int_{0}^{t}{\left(t-\tau \right)}^{-\alpha }\tau^{\beta }d\tau =\frac{\Gamma (1-\alpha )\Gamma (1+\beta )}{\Gamma (2-\alpha +\beta )}t^{1-\alpha +\beta }. \end{array}$$

*Therefore, according to the result in [29], the solution is*

(46) $$\begin{array}{c} Y^{f}\left(t\right)=y_{0}^{f}E_{\frac{1}{2}}\left(\frac{\Gamma \left(\frac{3}{2}\right)\Gamma \left(1+\beta \right)}{\Gamma (\frac{3}{2}+\beta )}t^{\frac{1}{2}+\beta }\right). \end{array}$$

*However, we find that $Y^{f}\left(t\right)$ defined in* (46) *is not the solution of Equation* (21).

*In fact, by using our obtained result in Lemma 3, the solution of Equation* (21) *is*

(47) $$\begin{array}{c} Y^{f}\left(t\right)=\sum_{i=0}^{\infty }R_{a}^{i}y_{0}^{f}=y_{0}^{f}E_{\frac{1}{2},1+2\beta ,2\beta }\left(t^{\frac{1}{2}+\beta }\right),t\in \left[0,T\right]. \end{array}$$

Next, we consider the solution of Equation (23).

**Lemma** **4.** *Let $\frac{1}{2}<H<1$ and σ∈C([0,T]). Then the solution of Equation* (23) *is*

(48) $$\begin{array}{c} Y^{s}\left(t\right)=y_{0}^{s}\exp\left(-H\int_{0}^{t}\tau^{2H-1}\sigma^{2}\left(\tau \right)d\tau +\int_{0}^{t}\sigma \left(\tau \right)dB_{H}\left(\tau \right)\right). \end{array}$$

**Proof.**  Let

(49) $$\begin{array}{c} Y^{s}\left(t\right)=y_{0}^{s}\exp\left(\int_{0}^{t}p_{1}\left(\tau \right)d\tau +\int_{0}^{t}p_{2}\left(\tau \right)dB_{H}\left(\tau \right)\right) \end{array}$$

be the solution of Equation (23). Then, it satisfies Equation (23); i.e.,

(50) $$\begin{array}{c} dY^{s}\left(t\right)=y_{0}^{s}\sigma \left(t\right)\exp\left(\int_{0}^{t}p_{1}\left(\tau \right)d\tau +\int_{0}^{t}p_{2}\left(\tau \right)dB_{H}\left(\tau \right)\right)dB_{H}\left(t\right). \end{array}$$

On the other hand, applying fractional Itô formula to $Y^{s}\left(t\right)$ in (49), we have

(51) $$\begin{array}{c} dY^{s}\left(t\right)=x_{0}\exp\left(\int_{0}^{t}p_{1}\left(\tau \right)d\tau +\int_{0}^{t}p_{2}\left(\tau \right)dB_{H}\left(\tau \right)\right)\left(p_{1}\left(t\right)+Ht^{2H-1}p_{2}^{2}\left(t\right)\right)dt+p_{2}\left(t\right)dB_{H}\left(t\right). \end{array}$$

Subtracting (50) from (51), we have

(52) $$\begin{array}{c} p_{2}\left(t\right)=\sigma \left(t\right),\;p_{1}\left(t\right)=-Ht^{2H-1}\sigma^{2}\left(t\right). \end{array}$$

Therefore, the solution of Equation (23) is

(53) $$\begin{array}{c} Y^{s}\left(t\right)=y_{0}^{s}\exp\left(-H\int_{0}^{t}\tau^{2H-1}\sigma^{2}\left(\tau \right)d\tau +\int_{0}^{t}\sigma \left(\tau \right)dB_{H}\left(\tau \right)\right). \end{array}$$

The proof of this lemma is completed. ☐

At this stage, we can establish the following theorem.

**Theorem** **1.** *Let a,b,σ∈C([0,T]), 0<α<1, and $\frac{1}{2}<H<1$. Then, the solution of Equation* (20) *is given by*

(54) $$\begin{array}{c} Y\left(t\right)=\exp\left(\int_{0}^{t}\left(b\left(\tau \right)-H\tau^{2H-1}\sigma^{2}\left(\tau \right)\right)d\tau +\int_{0}^{t}\sigma \left(\tau \right)dB_{H}\left(\tau \right)\right)\sum_{i=0}^{\infty }R_{a}^{i}y_{0}, \end{array}$$

*where $R_{a}$ is defined as* (28), *and $R_{a}^{i}$ denotes the i-times composition operator of $R_{a}$.*

We denote

(55) $$\begin{array}{c} \Phi \left(t\right)=\exp\left(\int_{0}^{t}b\left(\tau \right)d\tau -H\int_{0}^{t}\tau^{2H-1}\sigma^{2}\left(\tau \right)d\tau +\int_{0}^{t}\sigma \left(\tau \right)dB_{H}\left(\tau \right)\right)\sum_{i=0}^{\infty }R_{a}^{i}. \end{array}$$

One knows that Φ is the fundamental solution of Equation (20). In the following, we will show that Φ is invertible on [0,T] in an algebraic sense.

**Theorem** **2.** *Let* Φ *be the fundamental solution of Equation* (20). *Then* Φ *is invertible on [0,T], and its inverse is*

(56) $$\begin{array}{c} \Phi^{-1}=\exp\left(-\int_{0}^{t}\left(b\left(\tau \right)-H\tau^{2H-1}\sigma^{2}\left(\tau \right)\right)d\tau -\int_{0}^{t}\sigma \left(\tau \right)dB_{H}\left(\tau \right)\right)\sum_{i=0}^{\infty }{\left(-1\right)}^{i}R_{a}^{i}, \end{array}$$

*where $R_{a}$ is the operator defined as* (28).

**Proof.**  From Theorem 1, one knows that the following equation

(57) $$\begin{array}{c} dZ\left(t\right)=-\frac{1}{\Gamma (2-\alpha )}a\left(t\right)Z\left(t\right){\left(dt\right)}^{1-\alpha }-\left(b\left(t\right)-2Ht^{2H-1}\sigma^{2}\left(t\right)\right)Z\left(t\right)dt-\sigma \left(t\right)Z\left(t\right)dB_{H}\left(t\right),Z\left(0\right)=z_{0} \end{array}$$

has a unique solution $Z\left(t\right)=z_{0}\Psi \left(t\right)$, where Ψ(t) is the fundamental solution of Equation (57) given by

(58) $$\begin{array}{c} \Psi \left(t\right)=\exp\left(-\int_{0}^{t}\left(b\left(\tau \right)-H\tau^{2H-1}\sigma^{2}\left(\tau \right)\right)d\tau -\int_{0}^{t}\sigma \left(\tau \right)dB_{H}\left(\tau \right)\right)\sum_{i=0}^{\infty }{\left(-1\right)}^{i}R_{a}^{i}, \end{array}$$

and follows that

(59) $$\begin{array}{c} d\Psi \left(t\right)=-\frac{1}{\Gamma (2-\alpha )}a\left(t\right)\Psi \left(t\right){\left(dt\right)}^{1-\alpha }-\left(b\left(t\right)-2Ht^{2H-1}\sigma^{2}\left(t\right)\right)\Psi \left(t\right)dt-\sigma \left(t\right)\Psi \left(t\right)dB_{H}\left(t\right). \end{array}$$

Additionally, since Φ satisfies that

(60) $$\begin{array}{c} d\Phi \left(t\right)=\frac{1}{\Gamma (2-\alpha )}a\left(t\right)\Phi \left(t\right){\left(dt\right)}^{1-\alpha }+b\left(t\right)\Phi \left(t\right)dt+\sigma \left(t\right)\Phi \left(t\right)dB_{H}\left(t\right). \end{array}$$

Then, by the product rule, we have

(61) $$\begin{array}{c} d(\Phi \Psi )=\Psi (d\Phi )+\Phi (d\Psi )+d\Phi d\Psi =0. \end{array}$$

This implies that ΦΨ≡constant on t∈[0,T]. On the other hand, we note that Φ(0)Ψ(0)=1. Thus, Φ(t)Ψ(t)≡1 on t∈[0,T]. This implies that Φ is invertible on [0,T], and its inverse is Ψ. The proof is completed. ☐

### 3.2. Solution Representation for Linear Nonhomogeneous Case

In this subsection, we consider the solution of Equation (2). We use the variation of constants parameters to find a particular solution $Y_{p}$ of Equation (2). For this purpose, we define a random function

(62) $$\begin{array}{c} Y_{p}\left(t\right)=\Phi \left(t\right)c\left(t\right), \end{array}$$

where c(t) is an unknown random function with $c\left(0\right)=y_{0}$. Let us assume that $Y_{p}\left(t\right)$ is a solution of Equation (2).

By the product rule to $Y_{p}$, we have

(63) $$\begin{array}{c} dY_{p}\left(t\right)=d\Phi \left(t\right)c\left(t\right)+\Phi \left(t\right)dc\left(t\right)+d\Phi \left(t\right)dc\left(t\right). \end{array}$$

Additonally, since Φ is invertible, it has

(64) $$\begin{array}{c} dc\left(t\right)=\Phi^{-1}\left(dY_{p}\left(t\right)-d\Phi \left(t\right)c\left(t\right)-d\Phi \left(t\right)dc\left(t\right)\right). \end{array}$$

Furthermore, since $Y_{p}\left(t\right)$ is the solution of Equation (2) and Φ is the solution of Equation (20), we have

(65) $$\begin{array}{c} dc\left(t\right)=\Phi^{-1}\left(t\right)p\left(t\right){\left(dt\right)}^{1-\alpha }+\Phi^{-1}\left(t\right)q\left(t\right)dt+\Phi^{-1}\left(t\right)v\left(t\right)dB_{H}\left(t\right)-\Phi^{-1}d\Phi \left(t\right)dc\left(t\right). \end{array}$$

Additionally, since

(66) $$\begin{array}{c} d\Phi \left(t\right)dc\left(t\right)=2Ht^{2H-1}v\left(t\right)\sigma \left(t\right)dt. \end{array}$$

Therefore, we have

(67) $$\begin{array}{c} dc\left(t\right)=\Phi^{-1}\left(t\right)p\left(t\right){\left(dt\right)}^{1-\alpha }+\Phi^{-1}\left(t\right)\left(q\left(t\right)-2Ht^{2H-1}v\left(t\right)\sigma \left(t\right)\right)dt+\Phi^{-1}\left(t\right)v\left(t\right)dB_{H}\left(t\right), \end{array}$$

and

(68) $$\begin{array}{ccc} c(t) & = & c\left(0\right)+\int_{0}^{t}\Phi^{-1}\left(\tau \right)p\left(\tau \right){\left(d\tau \right)}^{1-\alpha }+\int_{0}^{t}\Phi^{-1}\left(\tau \right)\left(q\left(\tau \right)-2H\tau^{2H-1}v\left(\tau \right)\sigma \left(\tau \right)\right)d\tau \\ & + & \int_{0}^{t}\Phi^{-1}\left(\tau \right)v\left(\tau \right)dB_{H}\left(\tau \right). \end{array}$$

Thus, the solution Y(t) of Equation (2) is

(69) $$\begin{array}{ccc} Y(t) & = & \Phi \left(t\right)y_{0}+\int_{0}^{t}\Phi \left(t,\tau \right)p\left(\tau \right){\left(d\tau \right)}^{1-\alpha }+\int_{0}^{t}\Phi \left(t,\tau \right)\left(q\left(\tau \right)-2H\tau^{2H-1}v\left(\tau \right)\sigma \left(\tau \right)\right)d\tau \\ & + & \int_{0}^{t}\Phi \left(t,\tau \right)v\left(\tau \right)dB_{H}\left(\tau \right), \end{array}$$

where $\Phi \left(t,\tau \right)=\Phi \left(t\right)\Phi^{-1}\left(\tau \right)$, Φ and $\Phi^{-1}$ are defined as (55) and (56), respectively.

Based on the above analysis, we can establish the following theorem.

**Theorem** **3.** *Let a,p,q,v,σ∈C[0,T], 0<α<1 and $\frac{1}{2}<H<1$. Then the solution of Equation* (2) *is given by*

(70) $$\begin{array}{ccc} Y(t) & = & \Phi \left(t\right)y_{0}+\int_{0}^{t}\Phi \left(t,\tau \right)p\left(\tau \right){\left(d\tau \right)}^{1-\alpha }+\int_{0}^{t}\Phi \left(t,\tau \right)\left(q\left(\tau \right)-2H\tau^{2H-1}v\left(\tau \right)\sigma \left(\tau \right)\right)d\tau \\ & + & \int_{0}^{t}\Phi \left(t,\tau \right)v\left(\tau \right)dB_{H}\left(\tau \right), \end{array}$$

*where $\Phi \left(t,\tau \right)=\Phi \left(t\right)\Phi^{-1}\left(\tau \right)$*, Φ *and $\Phi^{-1}$ are defined as* (55) *and* (56), *respectively.*

## 4. Applications

In this section, we demonstrate some applications of our obtained results.

**Example** **1.** In this example, we consider a a mathematical model that can simulate the prices of financial instruments (e.g., stocks).

*Let (Ω,F,P) be a probability space, where* Ω *is called a sample space, F is a set of all events and possible statements about the prices on the market, and P is the usual probability measure. The price of an asset $Z_{t}$ in classical Black–Scholes model is assumed to follow Geometric Brownian motion given by*

(71) $$\begin{array}{c} dZ_{t}=\left(\mu +\frac{1}{2}\sigma^{2}\right)Z_{t}dt+\sigma Z_{t}dW_{t},Z_{0}=z_{0}, \end{array}$$

*where $W_{t}$ is the standard Brownian motion with respect to P, σ>0 is the diffusion parameter, and μ∈R is the drift.*

*The classical Black–Scholes model was certainly a breakthrough in the option pricing apparatus, because in the financial market, one needs to consider the influence of maturity time and the strike price on the financial derivatives or other factors. For these reasons, the Black–Scholes model with subdiffusion term is assumed to follow fractional Brownian motion given by [41]*

(72) $$\begin{array}{c} \frac{dZ_{t}}{dt}+aD_{t}^{\alpha }Z_{t}=\left(\mu +\frac{1}{2}\sigma^{2}\right)Z_{t}+\sigma Z_{t}\frac{dB_{H}\left(t\right)}{dt},Z_{0}=z_{0}, \end{array}$$

*where $B_{H}$ is the fractional Brownian motion with respect to P, $H\in (\frac{1}{2},1)$, a>0 is the subdiffusion parameter, σ>0 is the diffusion parameter, and μ∈R is the drift.*

For obtaining the solution of Equation (72), we divide three steps to solve it:

Step 1: According to (19), Equation (72) is equivalent to the following integral equation:

(73) $$\left\{\begin{array}{c} dZ_{t}=\frac{a}{\Gamma (2-\alpha )}Z_{t}{\left(dt\right)}^{1-\alpha }+\left(\mu +\frac{1}{2}\sigma^{2}\right)Z_{t}dt+\sigma Z_{t}dB_{H}\left(t\right),\\ Z_{0}=z_{0}. \end{array}\right.$$

So, we only need to solve (73). Furthermore, according to (21), (22), and (23), $Z_{t}$ can be expressed as $Z_{t}=Z_{t}^{f}Z_{t}^{d}Z_{t}^{s}$, where $Z_{t}^{f}$ is the solution of the following equation:

(74) $$\begin{array}{c} dZ_{t}^{f}=\frac{a}{\Gamma (2-\alpha )}Z_{t}^{f}{\left(dt\right)}^{1-\alpha },Z_{0}^{f}=z_{0}^{f}, \end{array}$$

$Z_{t}^{d}$ is the solution of the following equation:

(75) $$\begin{array}{c} dZ_{t}^{d}=\left(\mu +\frac{1}{2}\sigma^{2}\right)Z_{t}^{d}dt,Z_{0}^{d}=z_{0}^{d}, \end{array}$$

and $Z_{t}^{s}$ is the solution of the following equation:

(76) $$\begin{array}{c} dZ_{t}^{s}=\sigma Z_{t}^{s}dB_{H}\left(t\right),Z_{0}^{s}=z_{0}^{s}, \end{array}$$

and also $z_{0}^{f}z_{0}^{d}z_{0}^{s}=z_{0}$.

Step 2: Solve Equations (74)–(76), respectively. According to Lemma 3, the solution of Equation (74) is $Z_{t}^{f}=z_{0}^{f}E_{1-\alpha }\left(at^{1-\alpha }\right)$. According to Lemma 4, the solution of Equation (76) is $Z_{t}^{s}=z_{0}^{s}\exp\left(-\frac{\sigma^{2}t^{2H}}{2}+\sigma B_{H}\left(t\right)\right)$.

Step 3: According to Theorem 1, $Z_{t}$ is given by

(77) $$\begin{array}{c} Z_{t}=z_{0}E_{1-\alpha }\left(at^{1-\alpha }\right)\exp\left(-\frac{\sigma^{2}t^{2H}}{2}+\sigma B_{H}\left(t\right)+\left(\mu +\frac{1}{2}\sigma^{2}\right)t\right). \end{array}$$

**Example** **2.** *Consider the following fractional stochastic partial differential equation*

(78) $$\begin{array}{c} \frac{\partial U(x,t)}{\partial t}+D_{t}^{\alpha }U\left(x,t\right)=-k_{p_{1}}{\left(-▵\right)}^{\frac{p_{1}}{2}}U\left(x,t\right)-k_{p_{2}}{\left(-▵\right)}^{\frac{p_{2}}{2}}U\left(x,t\right)+U\left(x,t\right)\frac{dB_{H}\left(t\right)}{dt}, \end{array}$$

*with the nonhomogeneous Dirichlet boundary conditions*

(79) $$\begin{array}{c} U(0,t)=U(L,t)=0, \end{array}$$

*and the initial condition*

(80) $$\begin{array}{c} U(x,0)=\phi (x), \end{array}$$

*where (x,t)∈[0,L]×[0,T] (L and T are constants), 0<α<1, $0<p_{1}\leq 1$, $1<p_{2}\leq 2$, $\frac{1}{2}<H<1$, and φ(x) is a random function.*

According to Lemma 2, the eigenvalues $\lambda_{n}^{2}\;(n=1,2,\ldots )$ of the operator (−▵) with the homogeneous boundary conditions are $\lambda_{n}^{2}=n^{2}\pi^{2}/L^{2}$, and the corresponding eigenfunctions are $\varphi_{n}\left(x\right)=\sin\left(n\pi x/L\right)$, n=1,2,…. Then we set

(81) $$\begin{array}{c} U\left(x,t\right)=\sum_{n=1}^{\infty }U_{n}\left(t\right)\sin\left(n\pi x/L\right). \end{array}$$

Substituting (81) into (78) and (80) leads to the following equation:

(82) $$\begin{array}{c} \frac{dU_{n}\left(t\right)}{dt}+D_{t}^{\alpha }U_{n}\left(t\right)=-k_{p_{1}}\lambda_{n}^{p_{1}}U_{n}\left(t\right)-k_{p_{2}}\lambda_{n}^{p_{2}}U_{n}\left(t\right)+U_{n}\left(t\right)\frac{dB_{H}\left(t\right)}{dt}, \end{array}$$

with the initial condition

(83) $$\begin{array}{c} U_{n}\left(0\right)=\frac{2}{L}\int_{0}^{L}\phi \left(x\right)\sin\left(n\pi x/L\right)dx. \end{array}$$

By Theorem 1, the solution of Equation (82) with the initial condition (83) is

(84) $$\begin{array}{c} U_{n}\left(t\right)=U_{n}\left(0\right)\exp\left(\left(-k_{p_{1}}\lambda_{n}^{p_{1}}-k_{p_{2}}\lambda_{n}^{p_{2}}\right)t-\frac{t^{2H}}{H}+B_{H}\left(t\right)\right)E_{1-\alpha }\left(t^{1-\alpha }\right). \end{array}$$

Therefore, the solution of Equation (78) with the boundary conditions (79) and the initial condition (80) is

(85) $$\begin{array}{c} u\left(x,t\right)=\sum_{n=1}^{\infty }U_{n}\left(0\right)\exp\left(\left(-k_{p_{1}}\lambda_{n}^{p_{1}}-k_{p_{2}}\lambda_{n}^{p_{2}}\right)t-\frac{t^{2H}}{H}+B_{H}\left(t\right)\right)E_{1-\alpha }\left(t^{1-\alpha }\right)\sin\left(n\pi x/L\right). \end{array}$$

**Example** **3.** *Consider the following fractional stochastic partial differential equation*

(86) $$\begin{array}{c} \frac{\partial U(x,t)}{\partial t}+D_{t}^{\alpha }U\left(x,t\right)=-k_{p_{1}}{\left(-▵\right)}^{\frac{p_{1}}{2}}U\left(x,t\right)-k_{p_{2}}{\left(-▵\right)}^{\frac{p_{2}}{2}}U\left(x,t\right)+v\left(t\right)\frac{dB_{H}\left(t\right)}{dt}+f\left(x,t\right), \end{array}$$

*with the nonhomogeneous Dirichlet boundary conditions*

(87) $$\begin{array}{c} U(0,t)=U(L,t)=0, \end{array}$$

*and the initial condition*

(88) $$\begin{array}{c} U(x,0)=\psi (x), \end{array}$$

*where (x,t)∈[0,L]×[0,T] (L and T are constants), 0<α<1, $0<p_{1}\leq 1$, $1<p_{2}\leq 2$, $\frac{1}{2}<H<1$, and φ(x) is a random function.*

According to Lemma 2, the eigenvalues $\lambda_{n}^{2}\;(n=1,2,\ldots )$ of the operator (−▵) with the homogeneous boundary conditions are $\lambda_{n}^{2}=n^{2}\pi^{2}/L^{2}$, and the corresponding eigenfunctions are $\varphi_{n}\left(x\right)=\sin\left(n\pi x/L\right)$, n=1,2,…. Then we set

(89) $$\begin{array}{c} U\left(x,t\right)=\sum_{n=1}^{\infty }U_{n}\left(t\right)\sin\left(n\pi x/L\right),\;f\left(x,t\right)=\sum_{n=1}^{\infty }f_{n}\left(t\right)\sin\left(n\pi x/L\right). \end{array}$$

Substituting (89) into (86) and (88) leads to the following equation

(90) $$\begin{array}{c} \frac{dU_{n}\left(t\right)}{dt}+D_{t}^{\alpha }U_{n}\left(t\right)=-k_{p_{1}}\lambda_{n}^{p_{1}}U_{n}\left(t\right)-k_{p_{2}}\lambda_{n}^{p_{2}}U_{n}\left(t\right)+v\left(t\right)\frac{dB_{H}\left(t\right)}{dt}+f_{n}\left(t\right), \end{array}$$

with the initial condition

(91) $$\begin{array}{c} U_{n}\left(0\right)=\frac{2}{L}\int_{0}^{L}\psi \left(x\right)\sin\left(n\pi x/L\right)dx. \end{array}$$

By Theorem 3, the solution of Equation (86) with the initial condition (87) is

(92) $$\begin{array}{c} U_{n}\left(t\right)=\Phi \left(t\right)U_{n}\left(0\right)+\int_{0}^{t}\Phi \left(t,\tau \right)f_{n}\left(\tau \right){\left(d\tau \right)}^{1-\alpha }+\int_{0}^{t}\Phi \left(t,\tau \right)f_{n}\left(\tau \right)d\tau +\int_{0}^{t}\Phi \left(t,\tau \right)v\left(\tau \right)dB_{H}\left(\tau \right), \end{array}$$

where

(93) $$\begin{array}{c} \Phi \left(t\right)=\exp\left(-(k_{p_{1}}\lambda_{n}^{p_{1}}+k_{p_{2}}\lambda_{n}^{p_{2}})t\right)E_{1-\alpha }\left(t^{1-\alpha }\right), \end{array}$$

(94) $$\begin{array}{c} \Phi^{-1}\left(t\right)=\exp\left((k_{p_{1}}\lambda_{n}^{p_{1}}+k_{p_{2}}\lambda_{n}^{p_{2}})t\right)E_{1-\alpha }\left(-t^{1-\alpha }\right), \end{array}$$

and $\Phi \left(t,\tau \right)=\Phi \left(t\right)\Phi^{-1}\left(\tau \right)$. Therefore, the solution of Equation (86) with the boundary condition (87) and the initial condition (88) is

(95) $$\begin{array}{c} U\left(x,t\right)=\sum_{n=1}^{\infty }U_{n}\left(t\right)\sin\left(n\pi x/L\right), \end{array}$$

where $U_{n}\left(t\right)$ is defined in (92).

## 5. Conclusions

In this paper, we gave analytical solutions of multi-time scale fractional stochastic differential equations driven by fractional Brownian motions. We first decomposed the homogeneous multi-time scale fractional stochastic differential equation driven by fractional Brownian motion into independent differential subequations, and gave its analytical solution. Then, we used the variation of constants parameters to obtain the solution of the nonhomogeneous multi-time scale fractional stochastic differential equation driven by fractional Brownian motion. Finally, we demonstrated the applicability of our obtained results in solving FSDEs.

FSPDEs are an important class of differential equations. In this paper, we combined our obtained results about fractional stochastic ordinary differential equations and spectral representation technique to give the analytical solutions of some FSPDEs. In the future, we will investigate entropy analyses including permutation entropy, fractional permutation entropy, sample entropy, and fractional sample entropy with the help of our obtained analytical solutions in some practical problems. On the other hand, we plan to use the obtained analytical solutions of FSPDEs to assess the computational performance and accuracy of their numerical solutions which we will develop.
